# The Diabetes Care Project: an Australian multicentre, cluster randomised controlled trial [study protocol]

**DOI:** 10.1186/1471-2458-13-1212

**Published:** 2013-12-20

**Authors:** Matthew J Leach, Leonie Segal, Adrian Esterman, Caroline Armour, Robyn McDermott, Tim Fountaine

**Affiliations:** 1 School of Nursing & Midwifery, University of South Australia, North Terrace, Adelaide, South Australia 5000, Australia; 2 Health Economics & Social Policy Group, University of South Australia, North Terrace, Adelaide, South Australia 5000, Australia; 3 McKinsey & Company, Level 14, 1 Collins Street, Melbourne, VIC 3000, Australia; 4 SA/NT Data Link, University of South Australia, North Terrace, Adelaide, South Australia 5000, Australia; 5 McKinsey & Company, Level 35, 88 Phillip Street, Sydney, NSW 2000, Australia

**Keywords:** Cluster randomised controlled trial, Coordinated care, Diabetes mellitus, Economic evaluation, General practice, Primary care

## Abstract

**Background:**

Diabetes mellitus is an increasingly prevalent metabolic disorder that is associated with substantial disease burden. Australia has an opportunity to improve ways of caring for the growing number of people with diabetes, but this may require changes to the way care is funded, organised and delivered. To inform how best to care for people with diabetes, and to identify the extent of change that is required to achieve this, the Diabetes Care Project (DCP) will evaluate the impact of two different, evidence-based models of care (compared to usual care) on clinical quality, patient and provider experience, and cost.

**Methods/Design:**

The DCP uses a pragmatic, cluster randomised controlled trial design. Accredited general practices that are situated within any of the seven Australian Medicare Locals/Divisions of General Practice that have agreed to take part in the study were invited to participate. Consenting practices will be randomly assigned to one of three treatment groups for approximately 18 to 22 months: (a) control group (usual care); (b) Intervention 1 (which tests improvements that could be made within the current funding model, facilitated through the use of an online chronic disease management network); or (c) Intervention 2 (which includes the same components as Intervention 1, as well as altered funding to support voluntary patient registration with their practice, incentive payments and a care facilitator). Adult patients who attend the enrolled practices and have established (≥12 month’s duration) type 1 diabetes mellitus or newly diagnosed or established type 2 diabetes mellitus are invited to participate. Multiple outcomes will be studied, including changes in glycosylated haemoglobin (primary outcome), changes in other biochemical and clinical metrics, incidence of diabetes-related complications, quality of life, clinical depression, success of tailored care, patient and practitioner satisfaction, and budget sustainability.

**Discussion:**

This project responds to a need for robust evidence of the clinical and economic effectiveness of coordinated care for the management of diabetes in the Australian primary care setting. The outcomes of the study will have implications not only for diabetes management, but also for the management of other chronic diseases, both in Australia and overseas.

**Trial registration:**

Australian New Zealand Clinical Trials Registry (ACTRN12612000363886); World Health Organisation (U1111-1128-0481).

## Background

Diabetes mellitus is a chronic metabolic disorder that poses a significant health care problem in both developed and developing countries around the world, including Australia. An estimated 366 million people are affected by diabetes worldwide, and the incidence and prevalence of diabetes continues to rise, with more than 552 million people expected to be affected by the disorder by 2030—an increase of 51 percent [1]. People with diabetes are at risk of developing a range of complications, including chronic renal disease, diabetic eye disease and cardiovascular disease [2]. The impact of diabetes on the health system is considerable (in terms of both financial and human resources), with four percent of hospitalisations, 3.7 percent of general practitioner visits and 7.2 million prescriptions in Australia in 2009–10 attributed to the condition [3]. Direct and indirect health care costs related to the management of diabetes in Australia were estimated at AU$3.1 billion (excluding Commonwealth benefits) for type 2 diabetes (in 2001–02) [4], and at least $430 million for type 1 diabetes (in 2004–05) [5]. The chronicity and prevalence of diabetes, and the complications associated with the disorder, mean that diabetes will become an increasingly large burden on the health system, and on society, if new and more effective ways to manage it are not identified.

Australia has a unique opportunity to find better ways of caring for people with diabetes. Despite clear clinical objectives, fewer than fifty percent of people diagnosed with diabetes achieve the recommended targets for glycosylated haemoglobin, blood pressure and blood lipids [6-11]. Although care planning has been shown to result in substantial improvements in clinical and process outcomes for people with diabetes [12], almost eighty percent of GPs believe that the current care planning system involves too much red tape [13]. As a result, only eighteen percent of people with diabetes complete the recommended annual cycle of care [14], and approximately seventy percent do not have a multidisciplinary care plan at all [15].

To inform how best to care for people with diabetes, and to identify the extent of change that is required to achieve this, the Australian Government established the Diabetes Care Project (DCP)—a three-year, cluster randomised controlled trial that will test the impact of two different models of care (in comparison with usual care) on clinical quality and patient and provider experience. These models of care are designed to evaluate a number of evidence-based changes that the National Health and Hospital Reform Commission (NHHRC) [16] and the National Diabetes Advisory Group have identified as having the potential to improve the way care is organised and delivered. These changes include: (1) the introduction of new funding models, such as the NHHRC’s recommendation to link funding with a health care home (i.e., a single primary health care service that people with chronic disease would voluntarily register with to ‘help to coordinate, guide and navigate access to the right range of multidisciplinary health service providers’); [16] (2) offering financial incentives to improve the quality of health care services; [17] (3) facilitating improved communication and information-sharing across multidisciplinary health services through the use of electronic health records; (4) changing the way the primary health care team work together (e.g., by introducing a care facilitator and making case conferences easier to organise); [18] and (5) allowing practices to more easily review their performance and make any necessary adjustments.

Intervention 1 of the DCP tests improvements that could be made within the current funding model and focuses on information, communication and education. Intervention 2 includes the same components as Intervention 1, as well as altered funding to support voluntary patient registration with practices (‘health care homes’), incentive payments and funding for a care facilitator. The objective of the trial is to compare the impact of these two interventions on clinical quality and patient and provider experience, and to determine if the interventions allow care to be provided in more flexible ways. The DCP also seeks to determine if these interventions are economically sustainable and scalable nationally. The findings from this project will be used to make recommendations for health policy changes in Australia.

## Methods/Design

### Study design

The Diabetes Care Project is a pragmatic, cluster randomised controlled trial with three parallel arms. Clustering is at the level of the general practice. A cluster design is used to minimise contamination between intervention and control participants.

### Research objectives

The project is designed to: (1) evaluate the impact of the two interventions (in comparison with usual care) on clinical outcomes in adults with diabetes mellitus, patient quality of life, continuity of care, multidisciplinary collaboration and interaction, and patient and provider satisfaction and empowerment; (2) determine if the interventions allow care to be provided in more flexible ways; and (3) determine whether the interventions are scalable nationally and economically sustainable.

### Recruitment

**Figure 1:**
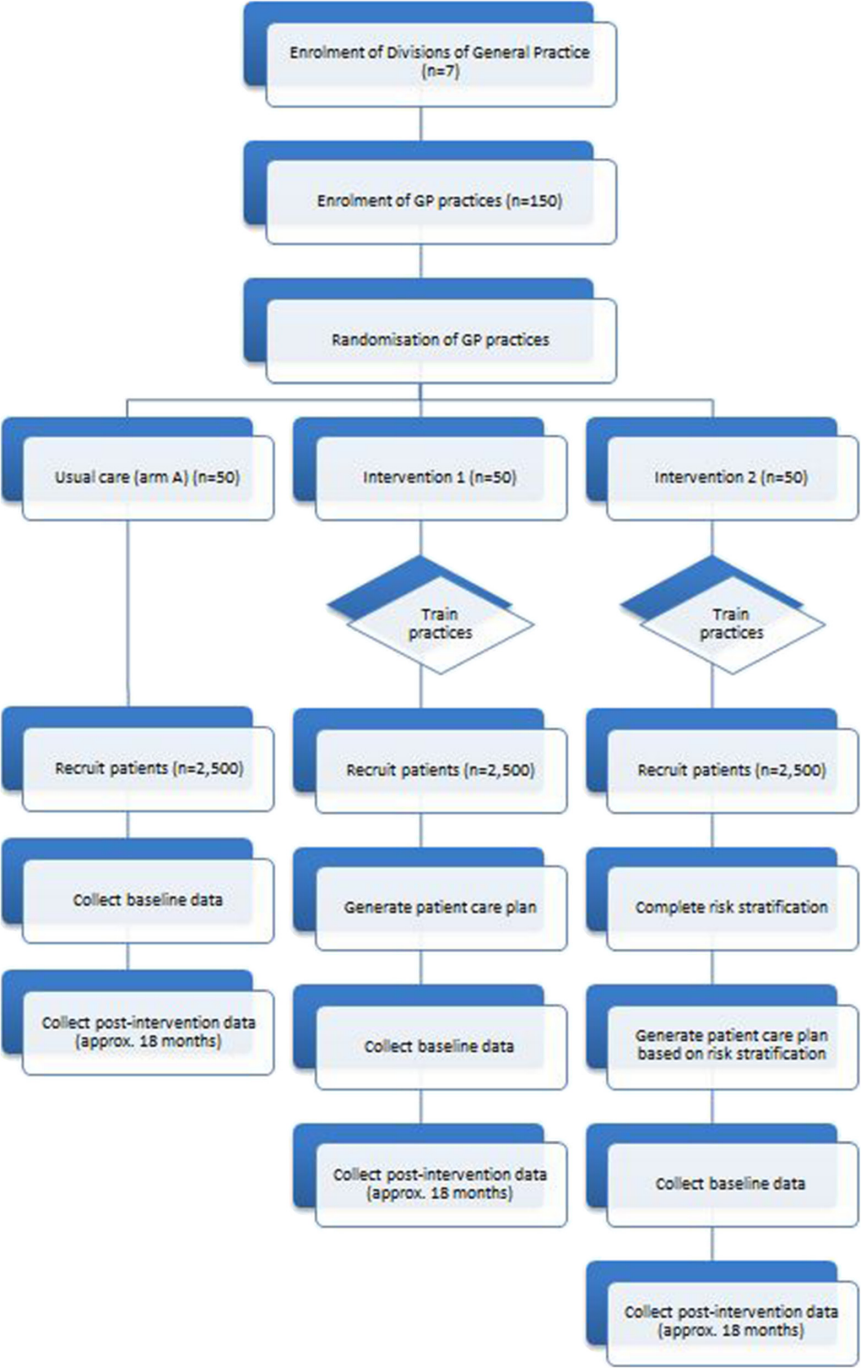
Flow chart of Diabetes Care Project.

Seven Medicare Locals/Divisions of General Practice (independent entities that are responsible for coordinating local primary health care services) [19] across the states of Queensland (n = 4), South Australia (n = 2), and Victoria (n = 1), Australia, are taking part in the trial. Prior to the commencement of the trial, each Medicare Local/Division of General Practice distributes study information sheets to all general practices (clusters) within their network and seeks expressions of interest to participate in the trial. Practices that express an interest in participating in the trial are enrolled in the project and randomly assigned to one of three treatment groups: control group, Intervention 1 or Intervention 2 (Figure 1). It is expected that fifty general practices will be enrolled in each group. Each enrolled practice is required to undergo training to familiarise themselves with the assigned intervention and study procedures. Medicare Locals/Divisions of General Practice that are assigned to Intervention 2 are supported by a ‘trial delivery and integration team’ to recruit and train care facilitators.

All eligible patients with diabetes are identified by the enrolled practice and the trial delivery and integration team and sent a GP-endorsed letter advertising the trial and actively seeking enrolments. A participant information sheet and all relevant consent forms are enclosed with this letter. A follow-up telephone call is made to all patients (either by the care facilitator, practice nurse or Medicare Local/Division of General Practice support person) to encourage participation and answer questions. After receiving informed consent, participants are asked to complete the baseline surveys and to meet with their GP to have their baseline metrics recorded. Participant recruitment commenced in April 2012, and patient participation will be completed by February 2014. The planned end date for the trial is June 2014.

### Randomisation

In four of the Medicare Locals/Divisions of General Practice, enrolled general practices are randomly allocated to the control group or Intervention 1 at a ratio of 1:2. In the other three Medicare Locals/Divisions of General Practice, practices are randomly assigned to the control group or Intervention 2 at a ratio of 1:2. A single type of intervention is tested in each Medicare Local/Division of General Practice as a result of two practical constraints: (1) care facilitators included in Intervention 2 are required to work with four or five practices within a single geographic area in order to work effectively; and (2) Medicare Locals/Divisions of General Practice would find it difficult to enrol and support practices using two different interventions, given that Intervention 1 and Intervention 2 comprise multiple components. Randomisation is applied after recruiting each group of three practices in a combined Medicare Local/Division of General Practice and Region stratum, where Region refers to urban or rural area. This helps to mitigate the potential bias created by testing only one intervention in each Medicare Local/Division of General Practice. Randomisation is applied separately for urban and rural practices within each Medicare Local/Division of General Practice because it is expected that service use and availability will differ by region. To approximate equality of sample sizes in each group, block randomisation is used with computer-generated, randomly permuted blocks of three. A researcher who is not involved in the implementation of the project and is blinded to the identity of the practices performs this task.

### Study population

#### Practice inclusion criteria

All practices situated within the Medicare Locals/Divisions of General Practice that have agreed to take part in the study (located in South Australia, Queensland and Victoria, Australia) are offered an opportunity to participate in the project, conditional on meeting the following criteria: (1) the practice has not indicated that they will not participate in trials; (2) practice software (GPs’ desktop application) is compatible with project software for data extraction purposes; (3) the practice meets the Royal Australian College of General Practitioners (RACGP) definition of ‘general practice’; (4) the practice is accredited (or registered for accreditation) against the current edition of the RACGP ‘Standards for general practices’; (5) the practice has current public liability insurance; and (6) all health professionals at the practice who will be providing care to enrolled patients are appropriately qualified and registered, and have current professional indemnity insurance. Most general practices meet these criteria.

#### Participant inclusion criteria

Adult patients of participating general practices are eligible to participate in the trial if they meet the following criteria: (1) they are aged 18 years or older; (2) they have established (≥12 months duration) type 1 diabetes mellitus or newly diagnosed or established type 2 diabetes mellitus; and (3) they have the capacity to provide informed consent to participate.

#### Participant exclusion criteria

Patients are excluded from the study if one or more of the following criteria are present: (1) they have a terminal illness with a life expectancy of less than two years; (2) they have dementia; (3) they are pregnant or planning to become pregnant in the next two years; and (4) they are participating in the Coordinated Veterans Care (CVC) program.

### Interventions

Each general practice (cluster) is randomly assigned to one of the three treatment arms. Participants in the control group are assigned ‘usual care’, meaning that practices will continue to provide care as usual.

Practices assigned to Intervention 1 experience two changes: access to an online chronic disease management network (cdmNet) and access to training and capability building. The cdmNet decision support service is instrumental in generating, updating and reviewing multidisciplinary care plans; facilitating communication between team members; sharing patient information; engaging patients in their care; and facilitating service payments. The care plans generated by cdmNet in Intervention 1 are pre-populated with the minimum care requirements for the general diabetes population, as informed by best practice guidelines and recommended treatments for any recognised comorbidities. CdmNet also generates population-level clinical status reports that allow GPs to proactively manage their population of people with diabetes enrolled in the trial. The training and capability building has three main functions: to assist practices in implementing the new model of care and cdmNet; to provide regular access to endocrinologists/diabetologists; and to provide a forum where practices can find out about new developments in diabetes care and share their experiences with other trial participants.

**Figure 2:**
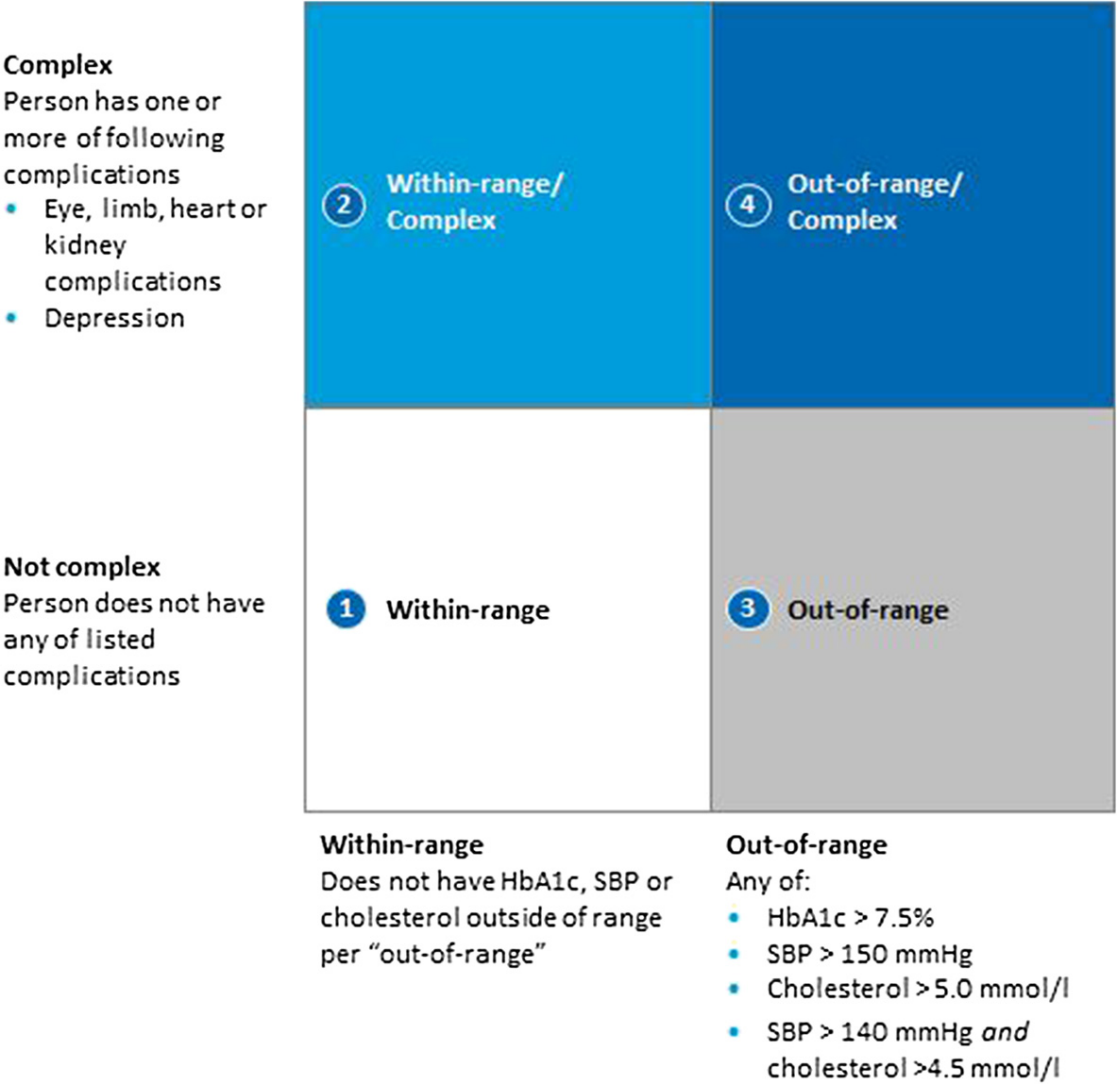
**Overview of risk stratification categories**^**#**^**.** HbA1c = Glycosylated haemoglobin; SBP = Systolic blood pressure. ^#^These risk stratification categories apply to participants with type 2 diabetes. Participants diagnosed with type 1 diabetes will be provided with the same level of resources as persons in the ‘out-of-range/complex’ category. Newly diagnosed patients will receive the support designated from their category plus additional support for introductory needs.

Practices assigned to Intervention 2 experience four changes: access to cdmNet; altered funding arrangements; the provision of a care facilitator; and access to training and capability building. Coordinated care is facilitated through the use of the cdmNet decision support service (as in Intervention 1), but with the addition of risk stratification. Patients are stratified into one of four risk categories based on whether they have complications associated with diabetes (e.g., peripheral neuropathy, background diabetic retinopathy, foot ulceration) and whether their diabetes is under control (uncontrolled diabetes requiring additional allied health services is defined as either HbA1c >7.5% [>58.5 mmol/mol], systolic blood pressure >150 mmHg, total serum cholesterol >5.0 mmol/l, or systolic blood pressure >140 mmHg plus total serum cholesterol >4.5 mmol/l). The four risk categories are characterised as follows: (1) not complex and within range; (2) complex and within range; (3) not complex and out of range; and (4) complex and out of range (see Figure 2). The care plan that is generated for the patient reflects the minimum care needs of individuals in their respective strata, as informed by best practice guidelines. Care in Intervention 2 is also coordinated through the provision of a full-time care facilitator (a tertiary-qualified health professional with experience in primary health care delivery), whose role is to assist the multidisciplinary team in delivering care in accordance with the agreed care plan. The care facilitator has a case load of approximately 300 to 400 patients across all four risk strata.

The altered funding arrangements that are tested in Intervention 2 comprise three components: flexible funding for general practices; pay-for-performance payments for general practices; and flexible funding for allied health professionals. Flexible funding for general practices provides upfront, lump sum funding for all patients who are voluntarily enrolled in the trial at a given practice, which is adjusted according to the risk of the patient population. This funding is used to fund care planning activities for those patients who require it (and is paid once a patient is enrolled rather than for completion of any specific activities) but it does not replace usual GP consultation fees covered by Medicare (Australia’s universal healthcare system). Pay-for-performance payments reward practices for improvements in patient clinical outcomes, meeting pre-defined levels of positive patient experience and completing pre-defined clinical processes. Flexible funding for allied health professionals is accessed on a fee-for-service basis, drawing on a risk-adjusted allowance for each patient. Participants in the control group, Intervention 1 and Intervention 2 will receive their respective care for a period of approximately 18 to 22 months (depending on when they are risk stratified).

### Outcome measures

#### Primary outcome

The primary endpoint is the difference in the change in HbA1c between groups. The study is powered to detect a difference of at least 0.25 percentage points in mean HbA1c between any two treatment groups. This difference is considered clinically significant on an intention-to-treat basis [20]. HbA1c data is collected by practice staff, as directed by the care plan, and recorded in cdmNet.

#### Secondary outcomes

Secondary outcomes include: changes in other biochemical and clinical metrics (specifically serum total cholesterol, serum triglycerides, serum low-density lipoprotein cholesterol, serum high-density lipoprotein cholesterol, estimated glomerular filtration rate and albumin:creatinine ratio, blood pressure, body mass index and waist circumference, as recorded in the GP patient record or patient’s local pathology laboratory); incidence of diabetes-related complications (specifically autonomic and peripheral neuropathy, peripheral arterial disease, diabetic foot ulceration, lower limb amputation, microalbuminuria, chronic kidney disease, proliferative and non-proliferative diabetic retinopathy, glaucoma, serious vision loss, acute state of severe uncontrolled diabetes requiring hospitalisation, myocardial infarction, stable and unstable angina, trans-ischaemic attack, cerebrovascular accident and sexual dysfunction); health-related quality of life (measured by the Assessment of Quality of Life - 4 Dimension [AQoL-4D] instrument); clinical depression (measured by the Patient Health Questionnaire); success of tailored care (assessed by the practitioner satisfaction survey, Patient Evaluation of the Quality of Diabetes Care survey, patient semi-structured interviews and practitioner focus groups); and economic sustainability (e.g., cost utility analysis). All outcomes are measured at baseline and will be measured again at the end of the trial (in approximately 18 to 22 months). Secondary outcome data will be sourced from patient and practitioner surveys (as outlined above), a patient diary, patient semi-structured interviews, practitioner focus groups, selected sections of the GP patient record imported into cdmNet, the Medicare Australia database (for Pharmaceutical Benefits Scheme and Medicare Benefits Scheme data), the National Diabetes Supply Scheme (NDSS) database, and hospital separation databases of the Queensland, Victorian and South Australian Departments of Health.

### Sample size calculation

A minimum sample size of 150 practices (or 50 practices per trial arm) and 3,750 patients (or 1,250 patients per trial arm) would allow a difference of 0.25 percentage points of HbA1c to be detected between any two trial arms with 80 percent power. The sample size is based a standard deviation of HbA1c of 1.4 percent, 20 percent attrition over 18 months, a design effect of 3.45 based on an ICC of 0.05 (correlation between baseline and 18-month HbA1c), and a two-sided alpha level set at 0.025 to adjust for pairwise comparisons.

### Statistical analysis

#### Main analysis

Descriptions of participants’ baseline characteristics (e.g., demographic data, comorbidities and clinical metrics) will be reported by treatment group. Categorical variables will be summarised by frequencies and percentages. Continuous variables will be summarised by means and SEMs, or non-parametric equivalents. All primary statistical analyses will be performed on an intention-to-treat basis. The continuous variable—HbA1c at approximately 18 to 22 months—will be compared between groups using a linear mixed effect model with adjustment for clustering and stratification status (Medicare Local/Division of General Practice and metro/rural practice location). The time/group interaction parameter will be used to formally test for an intervention effect. Secondary outcomes (excluding economic sustainability and value for money) will be analysed similarly. These findings will be presented in accordance with the reporting requirements outlined in the Consolidated Standards of Reporting Trials (CONSORT) statement - extension for cluster randomised trials [21].

#### Economic evaluation

Economic sustainability will be assessed by estimating the total mean per patient cost (including co-payment) and cost to government (separately for the Commonwealth and State governments) of patients in each of the three treatment groups. This will allow the DCP to estimate cost and budget impacts of the intervention models. Data will be sourced from Medicare Australia, State governments (for hospital in-patient, emergency and outpatient services), the patient diary and the NDSS. Depending on how well participants are matched across groups at baseline, some statistical adjustment may be necessary. Each person will be allocated to a diabetes disease stage (based on risk and morbidity profile), and the mean annual cost at each disease stage will be estimated across all three groups. The rate of progression in disease stage over the course of the study will be described for each patient and compared across groups to test for any between-group differences. If differences are detected, this will be used to model the downstream cost and budget implications of the three models of care.

A cost utility analysis will be conducted using the AQoL-4D to derive quality of life utility scores and to measure any change across the trial. Results for the three groups will be compared to identify any differential change in quality of life that can be multiplied by time to generate quality-adjusted life year (QALY) gain or loss for each intervention group. A quality of life utility score will be calculated for each disease stage (from persons in the disease stage), with a lower quality of life expected with more advanced disease. If a difference in the rate of disease progression is identified, this will be used to model the downstream impact on QALYs. The cost of implementing the model will be derived from trial administrative data and health care costs as described above. These costs will be combined with QALYs (within the trial and modelled beyond the trial) to estimate cost per QALY gain if it is found that the intervention is more expensive but delivers greater quality of life.

### Ethical considerations

The study protocol was approved by the human research ethics committees of the Department of Health and Ageing (Australian Government), Department of Human Services (Australian Government), Australian Institute of Health and Welfare (Australian Government), SA Department of Health (South Australian Government), Queensland Department of Health (Queensland Government), Department of Health Victoria (Victorian Government), and the Aboriginal Health Research Ethics Committee (Aboriginal Health Council of South Australia).

## Discussion

The DCP is designed to facilitate a robust yet pragmatic comparison of different, evidence-based models of care in order to evaluate how care can be improved for people with diabetes, and to determine the level of change that would be required to achieve this in the Australian context. Intervention 1 tests the extent to which current reforms (such as e-Health and clinical training initiatives) will improve care for people with diabetes. Intervention 2 tests the impact of altering primary care funding to create a ‘health care home,’ in which patients voluntarily register with a practice and are cared for by a multidisciplinary team coordinated by a care facilitator. Implementing the ‘health care home’ model of care (Intervention 2) at scale would involve substantial changes to the health system, and the DCP will therefore evaluate the incremental benefit of this intervention to ascertain if nationwide implementation is worthwhile.

A number of steps have been taken to ensure that this evaluation is rigorous and objective, and to increase the validity of the project’s final recommendations, including: introducing as much randomisation as is possible for this type of complex health reform; choosing populations to be representative of the Australian general practice landscape; and ensuring that the size of the trial will allow for testing of a range of different hypotheses (for example, whether the interventions work better in small or large practices and in urban or non-urban areas, and whether they affect people with more complicated diabetes and people with less complicated diabetes in different ways).

Two potential drawbacks of the trial design are that Medicare Locals/Divisions of General Practice only support one of the two interventions, and that patients are enrolled after practices have been randomised. It was necessary to retain both of these design features in order to make the trial workable. Firstly, requiring practices to support both interventions would have placed impossible demands on general practitioners, allied health professionals and Medicare Locals/Divisions of General Practice. The DCP’s approach means that Medicare Locals/Divisions of General Practice need only train practices in, and provide support for, one of the interventions, and any bias between Medicare Locals/Divisions of General Practice will be detected and corrected for by the DCP’s evaluation team. Secondly, postponing randomisation until patient enrolment was complete would have added a delay of six months (following initial sign up) for patients starting in the trial, and would have required control practices to be trained in the intervention in order for patients to give informed consent, thus confounding results.

The overall design of the trial is driven by the need to find practical and workable solutions to improving diabetes management in Australia that are informed by front-line experience. The trial has been developed through consultation with clinicians and patients during the design phase, and the project receives regular input from the national Diabetes Advisory Group, which comprises seventeen leaders of professional bodies representing various clinicians and people with diabetes. The consortium that is delivering the project consists of over twenty organisations (including Medicare Locals/Divisions of General Practice, state-based primary care organisations, state health departments and universities), and the project runs quarterly feedback forums with participating clinicians and patients.

The increasing global prevalence of diabetes, and the accompanying rise in complications and associated excess morbidity and mortality, suggest that the current approach to caring for people with diabetes can be improved. As the largest randomised controlled trial on the management of diabetes in Australia, the DCP has an opportunity to compare new, evidence-based approaches to diabetes management and to identify a model of care that improves health outcomes and patient and practitioner experiences, is scalable nationally and is economically sustainable. The outcomes of the study will have important implications not only for diabetes management, but also for the management of other chronic diseases, both in Australia and overseas.

## Competing interests

The authors declare that they have no competing interests.

## Authors’ contributions

All authors contributed to the conceptualisation, drafting and editing of the manuscript. All authors read and approved the final manuscript.

## Pre-publication history

The pre-publication history for this paper can be accessed here:

http://www.biomedcentral.com/1471-2458/13/1212/prepub
